# Increasing Obesity in Treated Female HIV Patients from Sub-Saharan Africa: Potential Causes and Possible Targets for Intervention

**DOI:** 10.3389/fimmu.2014.00507

**Published:** 2014-11-13

**Authors:** Claire L. McCormick, Arianne M. Francis, Kim Iliffe, Helen Webb, Catherine J. Douch, Mark Pakianathan, Derek C. Macallan

**Affiliations:** ^1^Clinical Infection Unit & Department of Genitourinary Medicine, St. George’s Healthcare NHS Trust, London, UK; ^2^Infection and Immunity Research Institute, St. George’s, University of London, London, UK

**Keywords:** HIV, body weight, body mass index, obesity, antiretroviral treatment, ethnology, HIV-associated lipodystrophy syndrome

## Abstract

**Objectives:** To investigate changing nutritional demographics of treated HIV-1-infected patients and explore causes of obesity, particularly in women of African origin.

**Methods:** We prospectively reviewed nutritional demographics of clinic attenders at an urban European HIV clinic during four one-month periods at three-yearly intervals (2001, 2004, 2007, and 2010) and in two consecutive whole-year reviews (2010–2011 and 2011–2012). Risk-factors for obesity were assessed by multiple linear regression. A sub-study of 50 HIV-positive African female patients investigated body-size/shape perception using numerical, verbal, and pictorial cues.

**Results:** We found a dramatic rise in the prevalence of obesity (BMI > 30 kg/m^2^), from 8.5 (2001) to 28% (2011–2012) for all clinic attenders, of whom 86% were on antiretroviral treatment. Women of African origin were most affected, 49% being obese, with a further 32% overweight (BMI 25–30 kg/m^2^) in 2012. Clinical factors strongly associated with obesity included female gender, black African ethnicity, non-smoking, age, and CD4 count (all *P* < 0.001); greater duration of cART did not predict obesity. Individual weight-time trends mostly showed slow long-term progressive weight gain. Investigating body-weight perception, we found that weight and adiposity were underestimated by obese subjects, who showed a greater disparity between perceived and actual adiposity (*P* < 0.001). Obese subjects targeted more obese target “ideal” body shapes (*P* < 0.01), but were less satisfied with their body shape overall (*P* = 0.02).

**Conclusion:** Seropositive African women on antiretroviral treatment are at heightened risk of obesity. Although multifactorial, body-weight perception represents a potential target for intervention.

## Introduction

When the HIV/AIDS pandemic emerged in the 1980’s wasting represented the major metabolic consequence of the disease (1) and was recognized as an AIDS-defining criterion. The revolution in HIV care resulting from the introduction of combination antiretroviral treatment (cART) has been matched by dramatic improvements in nutritional status (2), although wasting remains problematic in some settings (3). Early treatment regimes were, however, associated with other metabolic sequelae, most notably HIV-associated lipodystrophy syndrome (HALS). The visceral adiposity associated with HALS is of particular concern because of its association with insulin resistance, diabetes, and cardiovascular disease, effects which may be compounded by the effects of cART itself (4, 5). The prevalence of HALS has declined markedly in recent years with changing patterns of drug use (6) but has been replaced by today’s emerging metabolic problem, obesity (7–9). Obesity, which represents an entity distinct from visceral lipodystrophy (10), has recently been reported in several HIV-positive cohorts (2, 7, 8, 11, 12). There is evidence that black African ethnicity and female gender increase the risk of weight gain on cART (2, 11, 13, 14), although most studies regarding African ethnicity have focused on African-American populations (15, 16).

If the enhanced risk of obesity among HIV-infected women of African origin is a real effect, several plausible mechanisms might be proposed. First, genetics are clearly pivotal (17, 18). BMI has been linked to specific chromosomal loci in two separate African-American populations (19) and resistin gene polymorphisms contribute to hyperlipidemia and insulin-resistance on cART (20). Polymorphisms in uncoupling proteins are linked to ethnicity (21, 22) and, specifically, variants of uncoupling protein-3 with reduced basal metabolism in African-American women (23). Second, physiological adaptations occur with migration or environmental and dietary changes; adaptations to early-life limited nutrition may constitute maladaptations to later-life nutritional abundance (24, 25). More acute physiological adaptations occur with wasting syndromes; typically, on recovery an “overshoot” occurs with preferential recovery of adipose and delayed recovery of lean tissue in the early recovery phase, as demonstrated both in post-starvation re-feeding (26) and recovery from pulmonary tuberculosis (27).

Such genetic and physiologic determinants of body weight may, however, be less relevant in clinical practice for two reasons; first, they represent poor targets for intervention and, second, powerful cultural and ethnic drivers for body-weight change are superimposed upon and may over-ride them. Body image is a key determinant of “usual” weight and body-weight perception influences food intake. Widely held dogma suggests that women of African origin are likely to desire larger body sizes (28). Although evidence for this tenet is largely anecdotal, several studies support it. For example, Ugandan-born subjects in Britain rated heavier figures more positively than British natives (29), and African-Americans recorded greater satisfaction with body image and less anxiety regarding weight than comparable white American women (30–32). Such attitudes can also be found among HIV-infected women. In one study, more seropositive African-Americans wished to be “bigger” than did non-African-Americans (32 versus 15%) and more overweight participants perceived themselves as of “normal” weight (81 versus 50%) (28). Such views are not limited to urban African-American communities. Qualitative studies in disadvantaged communities in South Africa found that increased body size represented a marker of well-being (33); furthermore, two-thirds of adolescent women associated fatness with happiness and health (34). Not all authors endorse this simplistic paradigm; an urban Los Angeles study found no evidence that ethnicity influences preference for body shape or tolerance of obesity (35). If weight perception is a significant determinant, it represents a factor potentially amenable to intervention.

This paper reports a series of studies aiming, first, to test the hypothesis that obesity is an emerging clinical issue for HIV-positive patients on treatment (2); second, to investigate risk-factors for obesity in this population; and third, to explore how body-weight and body-shape perception relate to nutritional status and obesity among women of African origin.

## Materials and Methods

This study followed relevant institutional and national guidelines and regulations with the agreement of the Local Research Ethics Committee. The setting was a London (UK) tertiary-hospital HIV clinic.

To assess whether obesity is an emerging clinical issue for HIV-positive patients on treatment, clinical and nutritional demographic data were collected in two ways:

(i) Prospective one-month surveys, collecting nutritional and basic demographic data, were performed on all adult (≥ 18y) HIV-clinic attenders in the month of February every 3 years from 2001 to 2010. [Some data have previously been published (2).]
(ii) Two consecutive whole-year surveys were performed in 2010–2011 (*n* = 1166) and 2011–2012 (*n* = 1031) in which extensive clinical data, including nutritional data, were collected. The data collection form was modified between the first and the second one-year survey, so each survey was analyzed separately. Repeat visits and pregnant patients (including ≤3-months post-natal) were excluded. BMI was classified according to WHO descriptors: <18.5, wasted; 18.5–20, underweight; 20–25, normal; 25–30, overweight; >30, obese (36). In order to assess how obesity had arisen in those with the highest BMI values, weight recordings were retrieved from the hospital notes of 30 consecutive obese African women with adequately complete records (representing >180 patient-years of drug exposure). Trends were analyzed descriptively, as in analogous HIV-wasting studies (37), and by linear regression to derive a rate of weight change per year.

To investigate the risk-factors for obesity, we analyzed the large (*n* > 1000) 2010–2011 and 2011–2012 cohorts, relating BMI to clinical parameters (listed below) by simple and multiple-stepwise regression. Preceding one-month cohorts were not analyzed in this way because of their smaller size. For a comparator “normal” population, we used the “Health Survey for England” data set, which includes BMI data on 11,022 UK residents, 5,443 of whom are classified by ethnicity (38).

The relationship between body-weight/shape perception and obesity was assessed in 50 adult non-pregnant African women using a structured questionnaire. Subjects were recruited consecutively from clinic attenders willing to participate, regardless of BMI (hence both obese and non-obese patients were included). The questionnaire was produced in-house after piloting preliminary drafts and included questions on weight history, treatment, perceived links between treatment and weight change, background history (country of origin), ability to identify other major cardiovascular risk factors (smoking, diabetes, hypertension, hypercholesterolemia, and family history of stroke/heart disease), and level of satisfaction with current weight on a Likert scale. Current perception of body shape/body weight was assessed in three complementary ways: (1) numerically in kilograms: subjects rated their “usual” and “ideal” weights (normalized to BMI for between-subject comparisons); (2) textually: identifying their current nutritional status in terms of WHO descriptors (“wasted, underweight, normal, overweight, obese”); and (3) pictorially using figural stimuli: subjects identified current and ideal body shapes using images of known adiposity. Two published series of silhouettes were used: one (Series A) consisted of seven silhouettes corresponding to specified BMI’s; (39) the other (Series B), nine silhouettes of increasing adiposity, not calibrated by BMI (40). For body image scores, background comparator data were derived from published cohorts of 16,728 Caucasians (41) and 389 American women of mixed ethnicity (42).

Analysis of potential causes of obesity was framed around five putative hypotheses. Statistical comparisons were made by Fishers exact test and linear regression using *Prism* (GraphPad Software Inc., CA, USA) and *Sigmaplot* (Systat Software Inc., CA, USA).

## Results

### Time-trends in nutritional demographics

**Figure 1:**
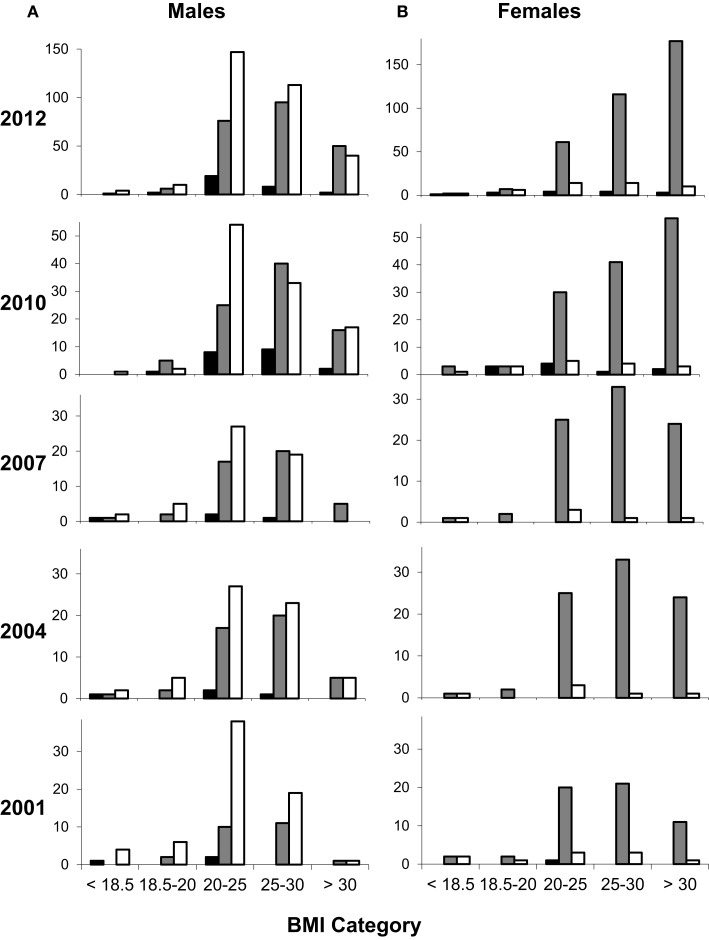
**Changing nutritional status of HIV-clinic attenders according to ethnicity and gender**. Values represent number of participants by gender [males **(A)** and females **(B)**], ethnicity (filled columns, Asian; shaded columns, black African; open columns, white Caucasian), and by BMI category: wasted (<18.5), undernourished (18.5–20), normal (20–25), overweight (25–30), and obese (>30 kg/m^2^). Data from 2001 to 2010 represent one-month prospective reviews, 2001 (*n* = 164; 96:68 male:female), 2004 (*n* = 204; 114:90), 2007 (*n* = 196; 109:87), and 2010 (*n* = 373; 213:160). Data from 2012 represent attenders over a whole year (*n* = 1031); similar data were obtained in 2011 (not shown). The small number of subjects in other ethnic groups is not shown for clarity.

One-month clinic datasets for nutritional demographics included 164, 204, 196, and 373 subjects in 2001, 2004, 2007, and 2010 respectively. Time-trends in BMI clearly showed an increasing prevalence of obesity over this period (Figure 1). This trend was most marked in women of black African descent; when each demographic group was considered separately (Figure S1 in Supplementary Material) this was the group most affected. In 2001, only 20% of black African women were obese, but this value increased progressively with each prospective three-yearly review, reaching 49% in 2012, a 2.5-fold increase in the prevalence of obesity over 10 years (Figure 1B). Male obesity also increased over the same time period, from 2 to 13%, although resulting levels of obesity were less marked (Figure 1A). This observation was not due to major changes in the overall demographics of patients attending the clinic (Figure S2 in Supplementary Material).

Individual weight-time graphs for obese African women, all of whom were on cART, revealed several distinct patterns (Figure S3 in Supplementary Material). The dominant pattern was one of slow progressive weight gain over several years (12/30); others showed early weight gain followed by stabilization (9/30), and some, obese pre-treatment, remained obese (7/30). The average weight trend over 183 person-years of follow-up was +0.62 kg/m^2^ year (Table 1). Rates were similar in patients on NNRTI- and PI-based regimens (means 0.64 and 0.59 kg/m^2^ year, respectively; *P* = 0.89; Table 1).

**Table 1:** **Weight trends of obese African women on cART by drug-class**.

Subject reference	NNRTI exposure (year)	Cumulative NNRTI effect (kg/m^2^)	NNRTI trend (kg/m^2^ year)	PI exposure (year)	Cumulative PI effect (kg/m^2^)	PI trend (kg/m^2^ year)
1	1.94	7.96	4.10	5.50	4.10	0.74
2	0.00			4.51	2.29	0.51
3	1.88	1.85	0.98	0.00		
4	0.00			5.32	1.08	0.20
5	15.24	3.48	0.23	1.11	2.80	2.52
6	0.00			2.63	2.80	1.06
7	2.33	−0.29	−0.13	0.00		
8	2.93	1.60	0.55	9.11	2.15	0.24
9	0.82	5.19	6.31	4.04	4.55	1.13
10	11.15	4.61	0.41	1.57	1.88	1.19
11	2.70	1.53	0.57	7.10	2.41	0.34
12	3.42	0.53	0.16	0.00		
13	5.86	−0.35	−0.06	0.00		
14	6.58	1.20	0.18	0.00		
15	7.21	3.64	0.50	0.00		
16	5.21	5.54	1.06	0.00		
17	0.90	2.55	2.83	1.33	2.54	1.92
18	1.50	2.65	1.76	0.00		
19	11.16	3.05	0.27	0.00		
20	13.02	4.21	0.32	0.00		
21	8.93	7.64	0.86	0.00		
22*	5.64	4.11	0.73	0.00		
23	1.11	0.11	0.10	0.00		
24	0.00			2.99	3.29	1.10
25	1.42	3.26	2.29	15.12	−0.59	−0.04
26	10.30	5.88	0.57	0.00		
27	1.00	0.66	0.67	0.00		
28	1.96	2.81	1.43	0.00		
29	0.48	0.59	1.23	1.15	6.96	6.05
30	2.07	7.39	3.58	0.00		
**Average**	**4.18**	**3.09**	**1.23**	**2.12**	**2.79**	**1.30**
**Sum**	**121.1**	**77.3**	**0.64**	**61.5**	**36.3**	**0.59**
**All treatments**	Exposure	Cumulative effect	Trend			
	182.6	113.6	0.62			

Analysis of weight trends from 30 obese women of black African origin. Trend was calculated by linear regression of BMI against time over the period of treatment exposure, excluding perinatal periods. Cumulative effect is the time-trend product. Average represents unweighted average of all episodes (*except #22, where almost the whole weight graph was perinatal). Sum represents the cumulative of all episodes and trend in this row is the sum cumulative effect divided by the cumulative exposure, i.e., a weighted average.

### Current nutritional demographics of HIV-clinic attenders

Since sequential one-month audits included only limited numbers of subjects, whole-year audits were performed in 2010–2011 and 2011–2012. As expected from the substantial overlap between cohorts (977 patients in both), results were similar. Results from the most recent cohort only are therefore presented including 1,031 subjects with median age 44 (range 19–83). Although overall similar numbers of men and women were included, more men were white Caucasian (314/599, 52%), whilst most women (84%) were of black African origin (363/432, including patients of black African extraction but more-recent Caribbean origin) (Table 2). Median CD4 was 500 cells/uL and 73% had an undetectable viral load.

**Table 2:** **Distribution of BMI groups by gender and race among clinic attenders 2011–2012**.

	Malnourished		Underweight		Normal		Overweight		Obese		Total	% of cohort by gender
	<18.5		18.5–20		20–25		25–30		>30			
	*n*	%	*n*	%	*n*	%	*n*	%	*n*	%	*n*	
**Women**											
Asian	0	0	0	0	2	33	3	50	1	17	6	1
Black African	2	1	7	2	55	17	108	33	158	48	330	76
Black Caribbean	0	0	0	0	5	19	6	22	16	59	27	6
Black Other	0	0	0	0	1	17	2	33	3	50	6	1
Chinese/Asian Other	1	11	3	33	2	22	1	11	2	22	9	2
White Caucasian	2	4	6	13	14	30	14	30	10	22	46	11
Other/mixed race	1	13	2	25	2	25	1	13	2	25	8	2
*Total women*	6	1	18	4	81	19	135	31	192	44	432	
**Men**											
Asian	0	0	1	7	7	47	6	40	1	7	15	3
Black African	0	0	4	2	47	28	80	47	39	23	170	28
Black Caribbean	0	0	2	4	25	53	12	26	8	17	47	8
Black Other	1	9	0	0	4	36	3	27	3	27	11	2
Chinese/Asian Other	0	0	1	6	12	75	2	13	1	6	16	3
White Caucasian	4	1	10	3	147	47	113	36	40	13	314	52
Other/mixed race	0	0	5	19	13	50	7	27	1	4	26	4
*Total men*	5	1	23	4	255	43	223	37	93	16	599	
**All**	11	1	41	4	336	33	358	35	285	28	1031	

*Number (*n*) and percentage (%) of HIV-clinic attenders within each BMI category for each defined major ethnic group. “White Caucasian” subjects were mostly of Western European origin*.

Assessment of BMI (Table 2) showed striking levels of obesity, most prevalent in women of black African origin, of whom 49% (177/363) were obese, with a further 32% (116/363) overweight. For females, black ethnicity gave a relative risk (RR) for obesity of 2.2 (95% confidence interval 1.4–3.6; *P* < 0.0001). In males, obesity was also associated with black ethnicity, 22% being obese (RR 1.9, 1.3–2.7; *P* = 0.001), although numbers were smaller (50/228). Conversely, wasting was uncommon in any sub-population; only 11/1,031 (1%) were wasted, and 52/1031 (5%) had a BMI of <20 kg/m^2^.

Having demonstrated a significant trend to obesity, predominantly affecting Black African women, we generated five possible explanatory hypotheses (framed as questions):

### Hypothesis 1: Is obesity in African women an effect of cART?

Interviews in the subgroup of 50 women from sub-Saharan Africa [principally Uganda (16) and Zimbabwe (10)] revealed that 48% felt their weight-gain was linked to their therapy (39% “definite” and 9% “probable”). However, the observed year-on-year increase in obesity was not associated with an increased proportion of patients receiving cART; 86% in the 2011–2012 cohort, compared with 83% in 2001. (Similar values pertained at intermediate 3-year reviews: 75, 86, and 92% in 2004, 2007, and 2010 respectively.) Furthermore, in the 2011–2012 cohort, there was no difference between the mean BMI of those on treatment and those not (27.5 ± 5.4 kg/m^2^, *n* = 884 versus 26.9 ± 5.2, *n* = 147, *P* = 0.21). Finally, there was no association between duration of cART and BMI in simple linear regression analysis (*r* = 0.020, *P* = 0.59, *n* = 884). These data do not therefore support a direct link between obesity and cART.

### Hypothesis 2: Is obesity the consequence of concomitant clinical factors?

In order to test this hypothesis, we performed stepwise multiple linear regression analyses relating clinically plausible predictors (age, gender, ethnicity, current smoking status, time since diagnosis, duration of cART, current viral load and CD4 count, glucocorticoid use, alcohol intake, diabetes mellitus, and hepatitis C) to current BMI for both recent whole-year reviews (analyzed separately as per section “Materials and Methods”). Very strong associations were found between BMI and gender, black African ethnicity, and smoking history (negatively) (Table 3). Age, CD4 count, and glucocorticoid use also emerged as important positive correlates. Duration of cART (taken as a linear variable) made a weak, but *negative*, contribution in the latter cohort (*P* = 0.013), and only after “time since diagnosis” was excluded from the model; the two are clearly collinear. Time since diagnosis, viral load, ethanol excess, diabetes, and hepatitis C did not add predictive power to the model. Route of exposure was considered but excluded because of collinearity. Gender and ethnicity thus emerge as the strongest predictors of BMI, whilst smoking reduces BMI.

**Table 3:** **Potential predictors or weight gain; correlates of high BMI**.

Factors	Units	*n* (%) or median (IQR)	*P*	Coeff	*r*
**2010–2011 Cohort**				
Model fit		1,166	<0.001		0.422
Female gender	*F* = 1	492 (42%)	<0.001	+2.07	0.291
Black African ethnicity	BA = 1	691 (59%)	<0.001	+1.73	0.290
Smoking	Yes = 1	265 (24%)	<0.001	−2.14	0.297
CD4 count	Cells/uL	500 (356, 678)	<0.001	+0.0025	0.113
Age	Years	43 (37, 49)	<0.001	+0.068	0.099
Not in model*					**Rank**
Duration cART	Years	5.9 (2.7, 9.6)	NS		3
Time since diagnosis	Years	6.6 (3.2, 11.5)	NS		2
Viral load	Copies/ml	0 (0, 56) 73% ND	NS		1
**2011–2012 Cohort**				
Model fit		1,031	<0.001		0.495
Female gender	*F* = 1	432 (42%)	<0.001	+2.85	0.314
Black African ethnicity	BA = 1	591 (57%)	<0.001	+2.07	0.328
Smoking	Yes = 1	216 (24%)	<0.001	−1.80	0.272
CD4 count	Cells/uL	500 (380, 706)	0.039	+0.0019	0.109
Age	Years	44 (38, 50)	<0.001	+0.0808	0.075
Glucocorticoid use	Yes = 1	72 (7.1%)	0.024	+1.85	0.092
Duration cART	Years	6.8 (3.6, 10.6)	0.002	−0.0004	0.020
Not in model*					**Rank**
History of alcohol excess	0–3	1 (0, 1)	NS		4
Time since diagnosis	Years	7.7 (4.1, 12.3)	NS		3
Diabetes mellitus	Yes = 1	47 (4.6%)	NS		2
Hepatitis C	Ab^+^ = 1	36 (4.0%)	NS		1

Data are from both 2010–2011 and 2011–2012 cohorts (*n* = 1,166 and 1,031, respectively; 977 subjects participated in both reviews). *P* values represent probability from backward stepwise multiple linear regression model after elimination of non-contributory variables, listed in reverse order of removal from the model (*P < 0.05); route of exposure was removed from the model *a priori* because of collinearity with gender and ethnicity. *r* values are from simple linear regression. Continuous variables are shown as median (first, third quartile); alcohol excess was graded 0–3 (none, occasional, moderate, heavy); dichotomous categorical values were attributed binary values (0 and 1) and are shown as number with attribute (% of recorded values).

### Hypothesis 3: Is obesity driven by an underestimation of weight?

**Figure 2:**
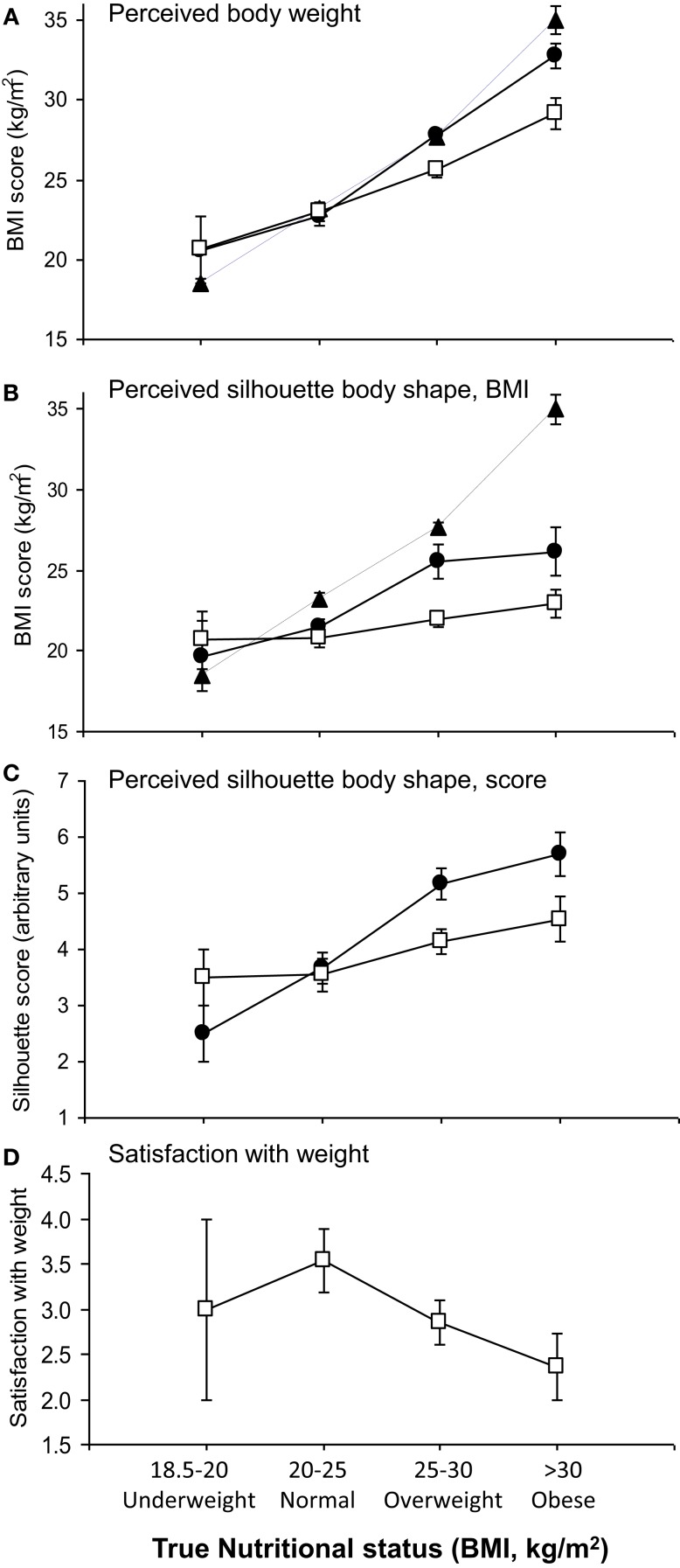
**Relationship of perceived and ideal body size to current nutritional status**. **(A)** Perceived body weight: True (filled triangles, dashed line), usual (filled circles), and ideal (open squares) body weight, expressed in kilograms (normalized to BMI) of patients according to current nutritional status category. “True” represents measured values, and “Usual” and “Ideal” were derived from questionnaire responses. **(B)** Perceived body shape from silhouettes corresponding to known BMI values (Series A) compared to true BMI (filled triangles). Data are shown for perceived “current” shape (filled circles) and “ideal” shape (open squares). **(C)** Perceived body shape from numbered silhouettes (Series B) expressed as “current” shape (filled circles) and “ideal” shape (open squares). **(D)** Subjects’ happiness with current body-weight scored from 1 to 5 (1, very unhappy; 2, unhappy; 3, not bothered; 4, happy; 5, very happy) according to current BMI category. All data shown are means ± 1 SEM.

Since it has been argued that weight perception differs in women of African origin (28), we investigated the contribution of body-weight perception to obesity in the subgroup of 50 African women. Most were first-generation immigrants (median duration in the UK 10 years), mostly (86%) from urban areas. Mean age was 40 years (range 20–60). Weight perception was explored using three complementary modalities. First, using weight in kilograms, subjects expressed a mean value for “usual” weight close to their current weight (Figure 2A, shown normalized to BMI). “Ideal” weights, however, differed between groups, correlating positively with BMI group (*r* = 0.70, *P* < 0.0001; Figure 2A). Thus, more obese subjects identified “ideal” body weights in the overweight range according to WHO criteria (mean BMI for ideal weights: 29.1 kg/m^2^). These values were significantly higher than the “ideal” values given by normal-weight women (23.0 kg/m^2^; *P* < 0.0001, one-way ANOVA).

Verbal descriptors yielded a similar pattern. Subjects usually described themselves with words corresponding to lower nutritional status categories than their WHO BMI category (Table 4). For example, 40% of obese subjects described themselves as “normal” weight, and 33% as “overweight”, whilst 47% of overweight patients used the “normal” designation.

**Table 4:** **Descriptions used by subjects to describe body weight according to nutritional status by BMI category**.

True BMI group		Perceived body weight									Totals
	Wasted		Underweight		Normal		Overweight		Obese	
	*n*	*%*	*n*	*%*	*n*	*%*	*n*	*%*	*n*	*%*	
Wasted (BMI > 18.5)	0	0	1	*50*	1	*50*	0	*0*	0	*0*	2
Underweight (BMI 18.5–20)	0	0	0	*0*	2	*100*	0	*0*	0	*0*	2
Normal (BMI 20–25)	0	0	1	*8*	8	*67*	3	*25*	0	*0*	12
Overweight (BMI 25–30)	0	0	1	*5*	9	*47*	8	*42*	1	*5*	19
Obese (BMI > 30)	0	0	0	*0*	6	*40*	5	*33*	4	*27*	15
All	0	0	3	*6*	26	*52*	16	*32*	5	*10*	*50*

*Responses from 50 black African women recruited consecutively from clinic attenders regardless of weight/BMI*.

Third, when body shape perception was analyzed using silhouettes corresponding to known BMI values [Series A (39)], obese subjects dramatically underestimated their current level of adiposity, identifying “current” body shape silhouettes corresponding to a mean BMI of 26.2 kg/m^2^, whereas their true mean BMI was 35.0 kg/m^2^ (*p* = 0.0002, Wilcoxon signed rank test). When asked about “ideal” body shape, both overweight and obese groups identified shapes less obese than their identified current shape with both silhouette series (Figures 2B,C; *P* < 0.01 by Wilcoxon signed rank test) but more obese subjects selected fatter “ideal” silhouettes than thinner subjects [Figure 2B (Series A), *r* = 0.33, *P* = 0.02; Figure 2C (Series B), *r* = 0.34, *P* = 0.02, Spearman]. The “ideal shape” selected by obese subjects corresponded to a mean BMI of 23 kg/m^2^, much less than their “ideal weight” (29.1 kg/m^2^), demonstrating a significant perceptual gap between body weight and body shape.

**Figure 3:**
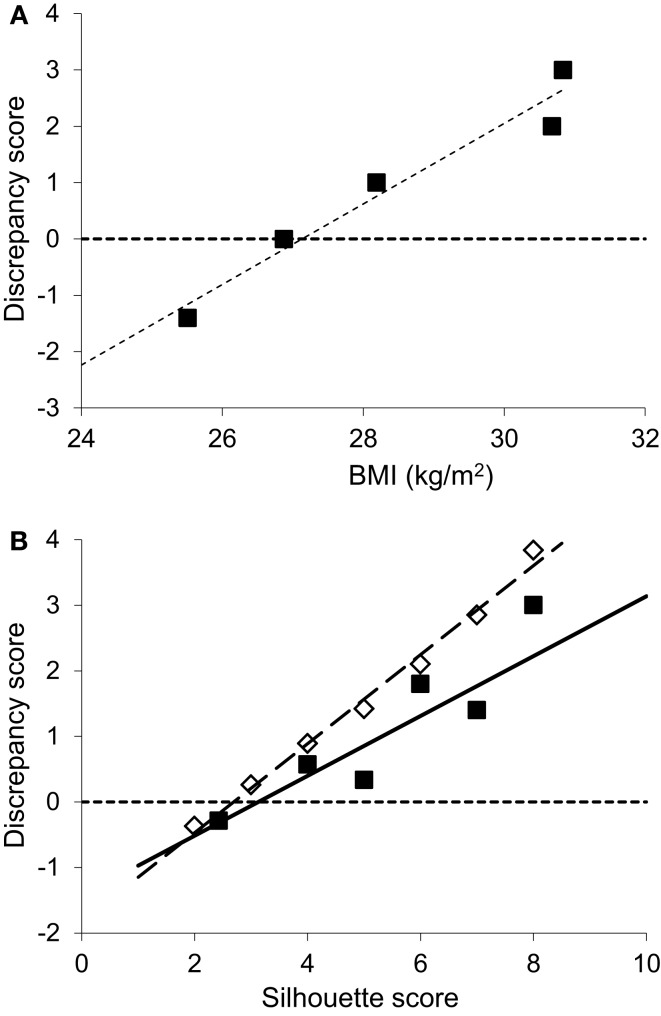
**Discrepancy between current and ideal body shape from body shape silhouettes**. **(A)** Discrepancy (current minus ideal) silhouette score groups, shown according to the corresponding current mean BMI for that group (groups with scores 1 and 2 were small so were conflated), showing higher discrepancy scores correspond to higher BMI’s (*r* = 0.37, intercept 26.9 kg/m^2^, *P* < 0.01, *n* = 47). **(B)** Filled squares: discrepancy scores by current silhouette score where higher silhouette score indicates greater adiposity (*r* = 0.61, *P* < 0.001, *n* = 47). Comparison is made with normative data (open diamonds) from a large Caucasian population (*n* = 16,728) (41).

The difference between current shape and ideal shape is customarily expressed as a “discrepancy score” (DS, = current – ideal shape) (41, 42), which can be taken as a surrogate marker for the drive to lose weight. As expected, and as widely observed in non-HIV populations, this score was greater in those with higher BMI. The intercept-value (DS = 0) corresponding to a threshold for body image dissatisfaction was 26.9 kg/m^2^ in this cohort (Figure 3A), a value in the “overweight” range, intermediate between published values for white urban Americans (24.6 kg/m^2^) and African-Americans (29.3 kg/m^2^) (42). Similarly, those with high DS scores identified themselves with fatter silhouettes (Figure 3B), but their drive to lose weight appeared “blunted” when compared to population-based normative data for Caucasians (41), each DS score corresponding to a silhouette about one score fatter than controls.

Thus all three modalities yielded similar conclusions that more obese women have heavier target weights but underestimate both their current level of obesity and the gap between current and “ideal” body weight, consistent with a reduced drive to lose weight.

### Hypothesis 4: Is obesity associated with a desire to be “fatter”?

Contrary to the hypothesis that African women are happy to be more obese, increased BMI was associated with a reduction in how happy subjects felt about their body weight, according to the satisfaction score attributed (Figure 2D; *r* = 0.32, *P* = 0.02, Spearman). The normal BMI group scored highest for satisfaction, 8/13 (62%) describing themselves as “very happy” or “happy” with their weight. In the obese group, only 4/14 (29%) responded with these scores, whilst 9/14 (64%) stated they were “unhappy” or “very unhappy” with their weight (*P* = 0.03 versus normal BMI group, chi-squared). This dissatisfaction was unrelated to concerns about cardiovascular risk; most women participating (35/50, 70%) were unaware of other major cardiovascular risk factors.

### Hypothesis 5: Is obesity the consequence of normalization to the population of origin?

**Figure 4:**
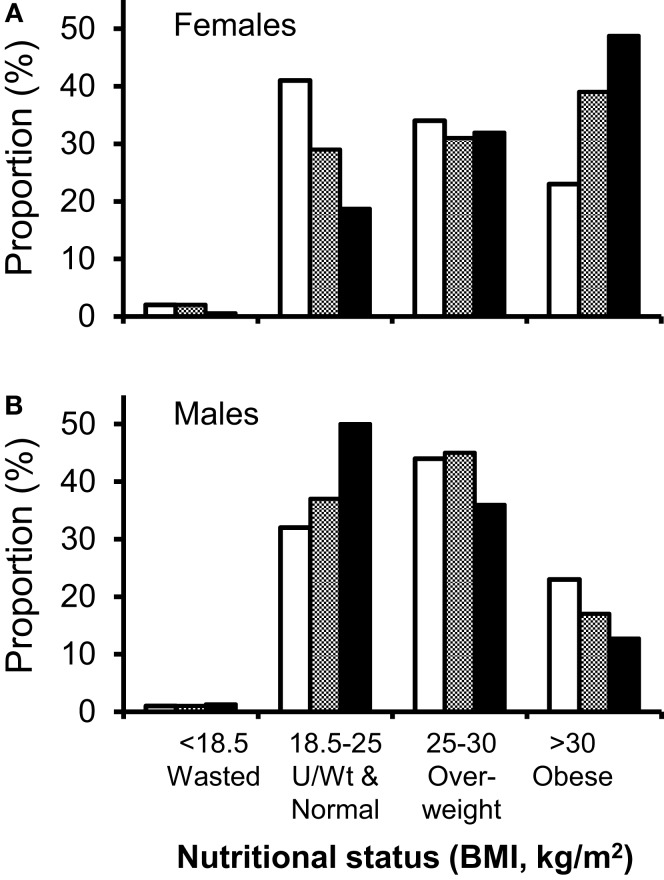
**Comparison of nutritional demographics in HIV-clinic attenders to the general population**. Distribution between different nutritional status groups for HIV-positive clinic attenders (solid bars) versus the general population (open and hatched bars). **(A)** Men, general population (open), black African men in general population (shaded), HIV-positive Caucasian males (solid bars), and **(B)** women, general population (open bars), black African women in general population (shaded), HIV-positive black African women (solid bars). Comparative data (HSE), expressed as percentage of each ethnic group, are from the Health Survey of England (*n* = 11,022; 5,443 classified by ethnic group, including 629 black Africans) (38). Note, HSE data did not give subgroup data for white Caucasian populations and were not subdivided at 20 kg/m^2^, so “underweight” and “normal” weight clinic attenders have been conflated to demonstrate comparable data.

An alternative hypothesis is that HIV-positive patients are simply “normalizing” to the nutritional demographics of their background population. We therefore compared our observations with the most recent comparable ethnically categorized data, the “2004 Health Survey for England: Health of Ethnic Minorities” (HSE) (38) which includes BMI data on 11,022 UK residents, 5,443 classified by ethnicity. Although this national survey reported high obesity rates in black African women (38% BMI > 30 kg/m^2^, versus 23% in the general female population, with a further 31% overweight), our obesity rates exceeded these values by about 10% (Figure 4A). Conversely men in our HIV-positive cohort had lower levels of obesity than the general population (Figure 4B). In men, black African ethnicity did not appear to favor obesity, either in our cohort (*P* = 0.56), or in HSE, where obesity rates in black African men were lower than the general population (17 versus 22%) (38). It might be argued that data from 2004 do not represent a sufficiently contemporaneous comparator; however, more recent national obesity data categorized by ethnicity could not be identified. In order to compensate for this time difference, we compared obesity rates in women in England of all ethnicities in 2004 with those in 2011. Rates increased from 23 to 26% (43) suggesting that time-trends in obesity in the general population, whilst increasing, are unlikely to explain the increase prevalence of obesity seen in our clinic cohort.

## Discussion

Our data strongly support the hypothesis that obesity is increasing in prevalence among HIV-positive patients on treatment, consistent with the paradigm that obesity is “…the latest epidemic” among HIV-infected individuals (7, 8). In our clinic-population, the effect was greatest among women of black African origin, 49% of whom were obese in this study. By contrast, wasting was very infrequent, although, of course, studying an outpatient cohort inevitably excluded acutely unwell in-patients and non-attenders in whom wasting may be more frequent.

We explored several plausible mechanistic hypotheses to explain the increasing prevalence of obesity in this population. Regression analysis identified gender, age and ethnicity as major correlates of this effect, together with a high CD4 count. Smoking was associated with lower BMI, as generally observed. Many women attributed their weight gain to antiretroviral therapy and individual weight-graphs (Figure S3 in Supplementary Material) suggest that weight gain occurs concurrently and progressively during successful treatment. However, this was not corroborated by analysis of duration of cART and BMI (where a weak correlation only emerged after adjusting for other parameters, and then the effect was negative). Not all collinearity could be controlled for; thus it is impossible to dissect duration of cART from era in which cART was started in this fixed time point cohort.

Such observations raise the question as to whether the weight-gain seen in this cohort [and others (2, 7, 8, 11, 12)] represents “normal” obesity, or is a variant of “HALS” (10). Certainly there is evidence that ethnicity alters the phenotypic expression of HALS, white race predisposing to lipoatrophy rather than fat accumulation (44). More detailed regional body composition data might clarify this issue, but were not collected here. Although changes in drug selection have resulted in a significantly reduced prevalence of HALS (6), our data suggest that weight-gain data should be monitored assiduously as the cumulative exposure to current agents increases and as new drugs are rolled out.

Gender and ethnicity are associated with obesity in the general population and our data follow similar trends. However, our findings suggest that the African women in this cohort have significantly “overshot” background levels of obesity (49 versus 38%), suggesting that regression to the norm is not an adequate explanation for the weight-gain seen in patients on treatment. The difficulties finding comparator data have been highlighted and it is hoped that future studies, such as further iterations of the Health Survey of England, will include ethnicity-specific data.

This study clearly has several limitations. First, the population studied has an implicit recruitment-bias toward those successfully adhering to treatment. Second, the silhouettes, although widely used and easy to understand, have coarse gradations, constraining choice, and are ethnically dependent; culturally relevant tools being developed may be more appropriate (45). Silhouette assessment represents a composite of both perceptual and attitudinal elements; more sophisticated tools such as digital morphing may allow additional insights (46). To test the nutritional-programing hypothesis, we included questions relating current obesity to birth-weight, but few respondents could answer these questions (data not shown). Collection of detailed body composition and dietary parameters would have enabled further hypothesis-testing. In terms of generalizability, we recognize the wide genetic diversity between different sub-Saharan African populations and the even greater differences, both genetic and cultural, between sub-Saharan and American-African populations, in whom European ancestry may contribute to obesity; (47) these two groups cannot be conflated into a single category. Reanalysis, excluding Caribbean patients from the category “of-African-origin” did not affect our results, so we believe our results describe a generalizable phenomenon, consistent with observations in other populations (2, 7, 8, 11, 12).

Since the major factors associated with this phenomenon, ethnicity, age and gender, are not amenable to intervention, we sought to identify factors which were. Exploring the contribution of body-weight perception to obesity revealed four major themes. First, obese black African HIV-positive women tended to underestimate current levels of adiposity (this was true for all three modalities tested: numerical weight, word descriptors and body shape silhouettes), similar to a South African cohort (48). Second, obese subjects defined higher “ideal” weights than their thinner counterparts. Third, there was a disparity between weight and shape: obese subjects tended to underestimate the weight loss (in kg) required to achieve a certain body shape. Fourth, disparity scores, which reflect the drive to lose weight, were lower in our population than comparator Caucasian populations.

Thus perceptions of obesity/adiposity may tend to promote or maintain weight-gain, but this is not the same as a desire to be fatter; indeed we found the converse was true. Greater adiposity was associated with lower, not higher, levels of satisfaction with current weight; obese patients in this cohort wanted to be thinner than their current weight. Fear of weight loss was not specifically mentioned in interviews but over half of the women surveyed had experienced profound prior illness-associated weight loss. Awareness of cardiovascular risk was low among this group of women and thus not a major motivator for weight loss; this may not be inappropriate as the average predicted 10-year CVD risk (49) for individuals in the obese group (including cholesterol and blood pressure, recorded but not presented here) was only 1.6%.

In conclusion, obesity appears to be a major developing problem among HIV-positive people on treatment, especially women of African origin. Although antiviral therapy was perceived as a contributing factor, we found little evidence to support this hypothesis. Rather we found evidence for a high target “ideal” weight, less dissatisfaction with a larger body shape, and poor awareness of cardiovascular risk in this cohort. These are important factors because they may be amenable to intervention. Although perceptions and attitudes may be difficult to influence, improved patient education and dietetic advice could seek to increase awareness of the propensity to and the risks of obesity. Even small changes may be worthwhile; in other settings (e.g. type-2 diabetes), minor reductions in weight can dramatically improve metabolic status (50).

## Author Contributions

Claire L. McCormick performed data collection and analysis; reviewed and approved final manuscript. Arianne M. Francis performed data collection and analysis; helped draft the manuscript. Kim Iliffe helped design the study; performed data collection and analysis; reviewed and approved final manuscript. Helen Webb recruited patients and performed data collection; reviewed and approved final manuscript. Catherine J. Douch performed data collection and analysis; reviewed and approved final manuscript. Mark Pakianathan helped design the study and oversaw data collection; reviewed and approved final manuscript. Derek C. Macallan conceived and designed the study, performed data analysis and drafted and edited the manuscript.

## Conflict of Interest Statement

The authors declare that the research was conducted in the absence of any commercial or financial relationships that could be construed as a potential conflict of interest.

## Supplementary Material

The Supplementary Material for this article can be found online at http://www.frontiersin.org/Journal/10.3389/fimmu.2014.00507/abstract
