# Supplementary material for: Increasing Obesity in Treated Female HIV Patients from Sub-Saharan Africa: Potential Causes and Possible Targets for Intervention
Source: Front Immunol. 2014 Nov 13;5:507. doi: 10.3389/fimmu.2014.00507 (PMC4230180; doi:10.3389/fimmu.2014.00507)
Supplement: Supplementary file 1 [file Presentation1.PPTX]

## Slide 1
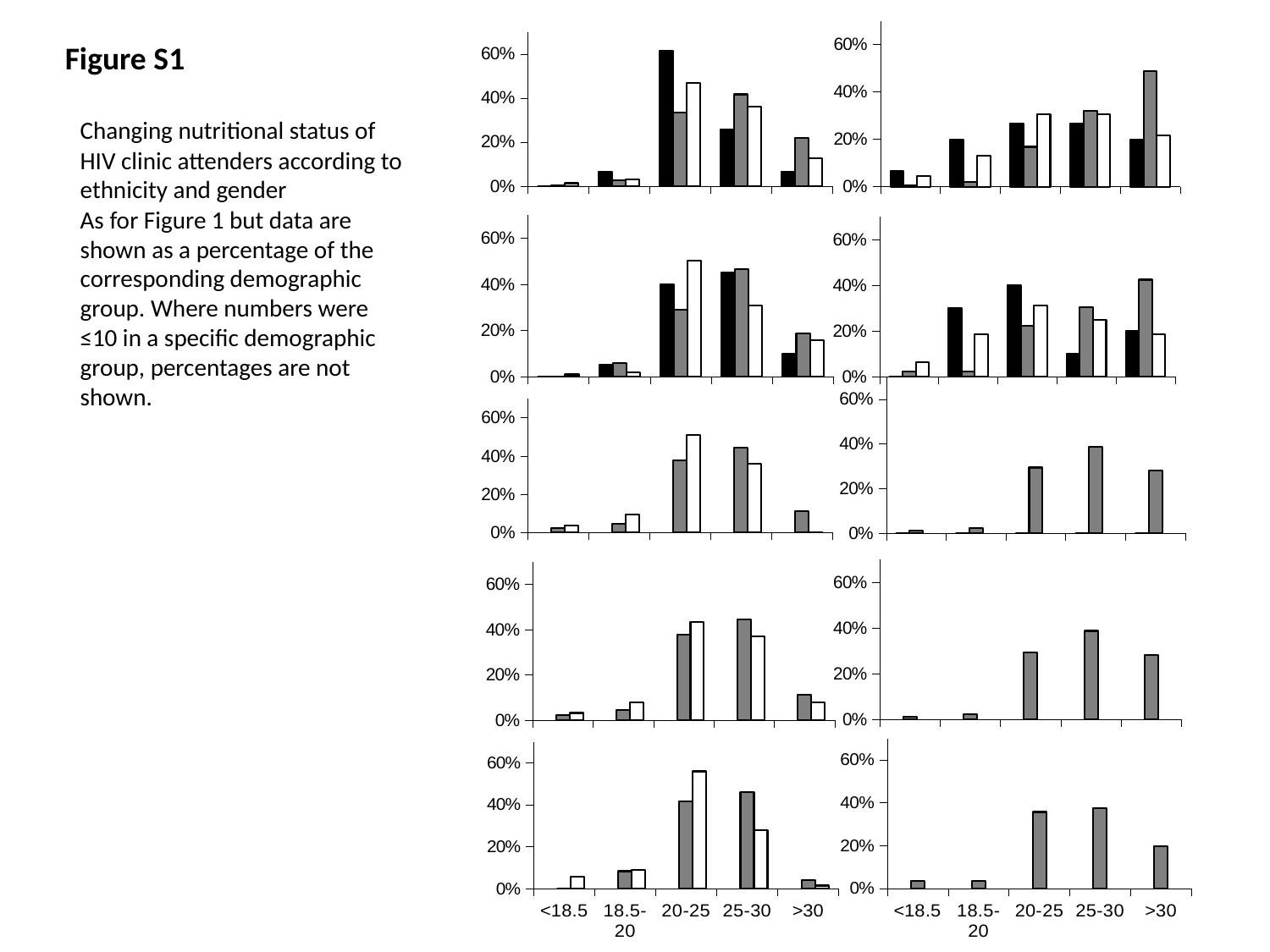

### Chart
| Category | | | |
|---|---|---|---|
| < 18.5 | 0.06666666666666667 | 0.005509641873278237 | 0.043478260869565216 |
| 18.5-20 | 0.2 | 0.01928374655647383 | 0.13043478260869565 |
| 20-25 | 0.26666666666666666 | 0.16804407713498623 | 0.30434782608695654 |
| 25-30 | 0.26666666666666666 | 0.31955922865013775 | 0.30434782608695654 |
| > 30 | 0.2 | 0.48760330578512395 | 0.21739130434782608 |
### Chart
| Category | | | |
|---|---|---|---|
| < 18.5 | 0.0 | 0.0043859649122807015 | 0.012738853503184714 |
| 18.5-20 | 0.06451612903225806 | 0.02631578947368421 | 0.03184713375796178 |
| 20-25 | 0.6129032258064516 | 0.3333333333333333 | 0.4681528662420382 |
| 25-30 | 0.25806451612903225 | 0.4166666666666667 | 0.35987261146496813 |
| > 30 | 0.06451612903225806 | 0.21929824561403508 | 0.12738853503184713 |Figure S1
Changing nutritional status of HIV clinic attenders according to ethnicity and gender
As for Figure 1 but data are shown as a percentage of the corresponding demographic group. Where numbers were ≤10 in a specific demographic group, percentages are not shown.
### Chart
| Category | Asian | Black | White Caucasian |
|---|---|---|---|
| <18.5 | 0.0 | 0.0 | 0.009345794392523364 |
| 18.5-20 | 0.05 | 0.05813953488372093 | 0.018691588785046728 |
| 20-25 | 0.4 | 0.29069767441860467 | 0.5046728971962616 |
| 25-30 | 0.45 | 0.46511627906976744 | 0.308411214953271 |
| >30 | 0.1 | 0.18604651162790697 | 0.1588785046728972 |
### Chart
| Category | Asian | Black | White Caucasian |
|---|---|---|---|
| <18.5 | 0.0 | 0.022388059701492536 | 0.0625 |
| 18.5-20 | 0.3 | 0.022388059701492536 | 0.1875 |
| 20-25 | 0.4 | 0.22388059701492538 | 0.3125 |
| 25-30 | 0.1 | 0.30597014925373134 | 0.25 |
| >30 | 0.2 | 0.4253731343283582 | 0.1875 |
### Chart
| Category | Asian | Black | White |
|---|---|---|---|
| < 18.5 | 0.0 | 0.011764705882352941 | None |
| 18.5-20 | 0.0 | 0.023529411764705882 | None |
| 20-25 | 0.0 | 0.29411764705882354 | None |
| 25-30 | 0.0 | 0.38823529411764707 | None |
| > 30 | 0.0 | 0.2823529411764706 | None |
### Chart
| Category | Asian | Black | White |
|---|---|---|---|
| < 18.5 | None | 0.022222222222222223 | 0.03773584905660377 |
| 18.5-20 | None | 0.044444444444444446 | 0.09433962264150944 |
| 20-25 | None | 0.37777777777777777 | 0.5094339622641509 |
| 25-30 | None | 0.4444444444444444 | 0.3584905660377358 |
| > 30 | None | 0.1111111111111111 | 0.0 |
### Chart
| Category | Asian | Black | White |
|---|---|---|---|
| 0 | None | 0.011764705882352941 | None |
### Chart
| Category | Asian | Black | White |
|---|---|---|---|
| 0 | None | 0.022222222222222223 | 0.03225806451612903 |
### Chart
| Category | Asian Other | Black | White |
|---|---|---|---|
| <18.5 | None | 0.03571428571428571 | None |
| 18.5-20 | None | 0.03571428571428571 | None |
| 20-25 | None | 0.35714285714285715 | None |
| 25-30 | None | 0.375 | None |
| >30 | None | 0.19642857142857142 | None |
### Chart
| Category | Asian Other | Black | White |
|---|---|---|---|
| <18.5 | None | 0.0 | 0.058823529411764705 |
| 18.5-20 | None | 0.08333333333333333 | 0.08823529411764706 |
| 20-25 | None | 0.4166666666666667 | 0.5588235294117647 |
| 25-30 | None | 0.4583333333333333 | 0.27941176470588236 |
| >30 | None | 0.041666666666666664 | 0.014705882352941176 |

## Slide 2
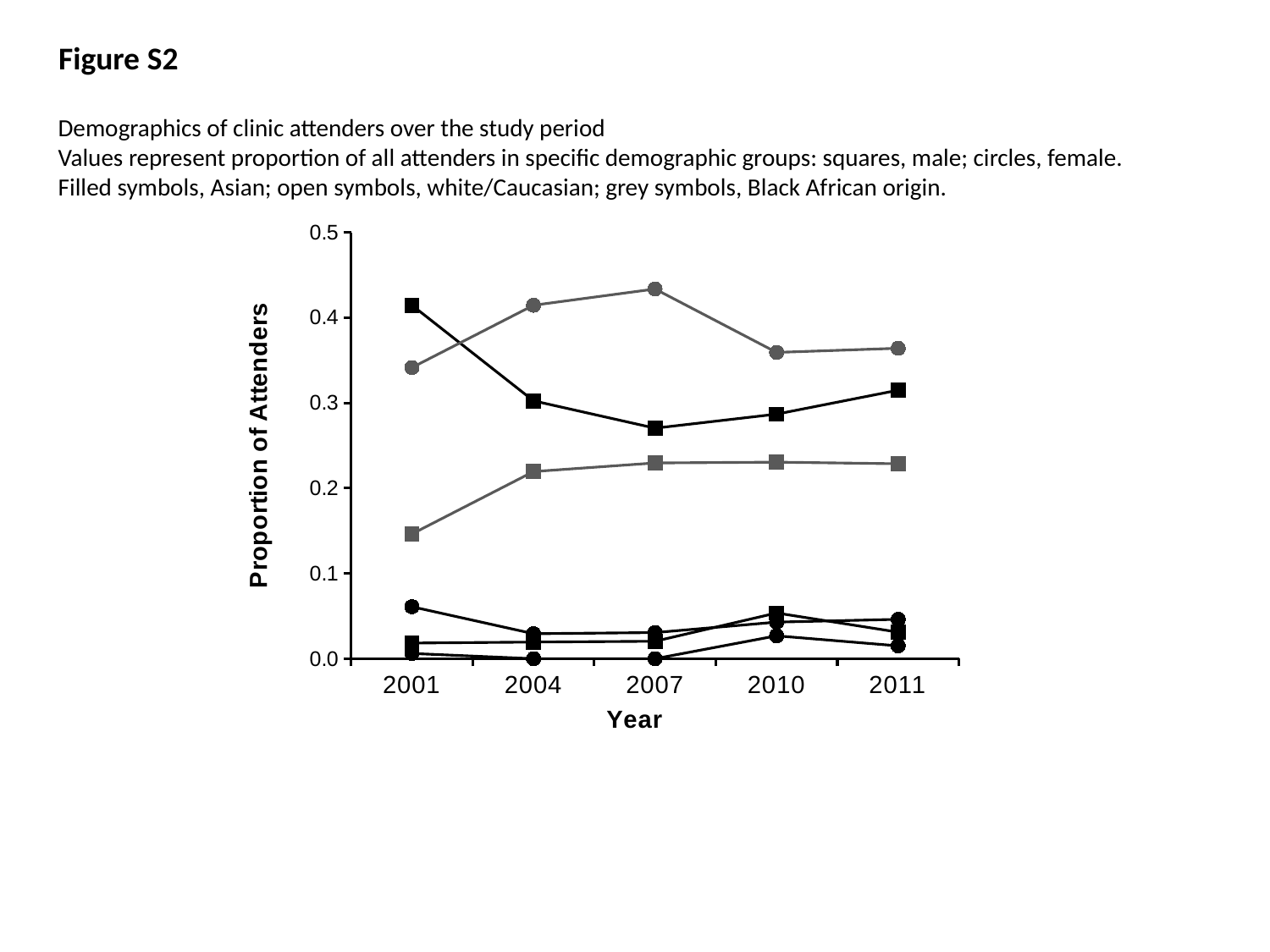

Figure S2
Demographics of clinic attenders over the study period
Values represent proportion of all attenders in specific demographic groups: squares, male; circles, female. Filled symbols, Asian; open symbols, white/Caucasian; grey symbols, Black African origin.
### Chart
| Category | | | | | | |
|---|---|---|---|---|---|---|
| 2001 | 0.018292682926829267 | 0.14634146341463414 | 0.4146341463414634 | 0.34146341463414637 | 0.06097560975609756 | 0.006097560975609756 |
| 2004 | 0.01951219512195122 | 0.21951219512195122 | 0.3024390243902439 | 0.4146341463414634 | 0.02926829268292683 | 0.0 |
| 2007 | 0.02040816326530612 | 0.22959183673469388 | 0.27040816326530615 | 0.4336734693877551 | 0.030612244897959183 | 0.0 |
| 2010 | 0.05361930294906166 | 0.23056300268096513 | 0.2868632707774799 | 0.35924932975871315 | 0.04289544235924933 | 0.02680965147453083 |
| 2011 | 0.031093279839518557 | 0.22868605817452356 | 0.3149448345035105 | 0.3640922768304915 | 0.04613841524573721 | 0.015045135406218655 |

## Slide 3
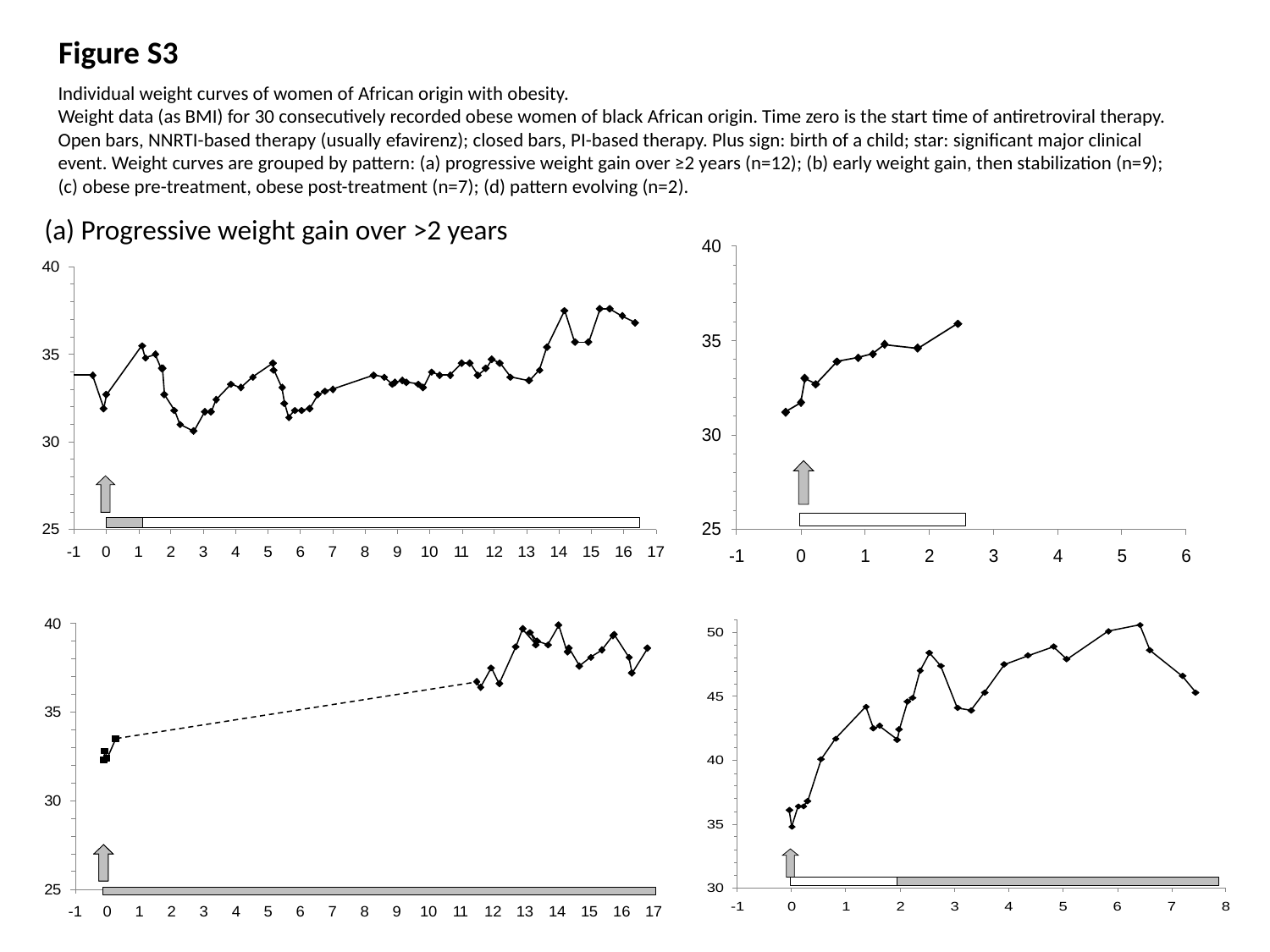

Figure S3
Individual weight curves of women of African origin with obesity.
Weight data (as BMI) for 30 consecutively recorded obese women of black African origin. Time zero is the start time of antiretroviral therapy. Open bars, NNRTI-based therapy (usually efavirenz); closed bars, PI-based therapy. Plus sign: birth of a child; star: significant major clinical event. Weight curves are grouped by pattern: (a) progressive weight gain over ≥2 years (n=12); (b) early weight gain, then stabilization (n=9); (c) obese pre-treatment, obese post-treatment (n=7); (d) pattern evolving (n=2).
(a) Progressive weight gain over >2 years

## Slide 4
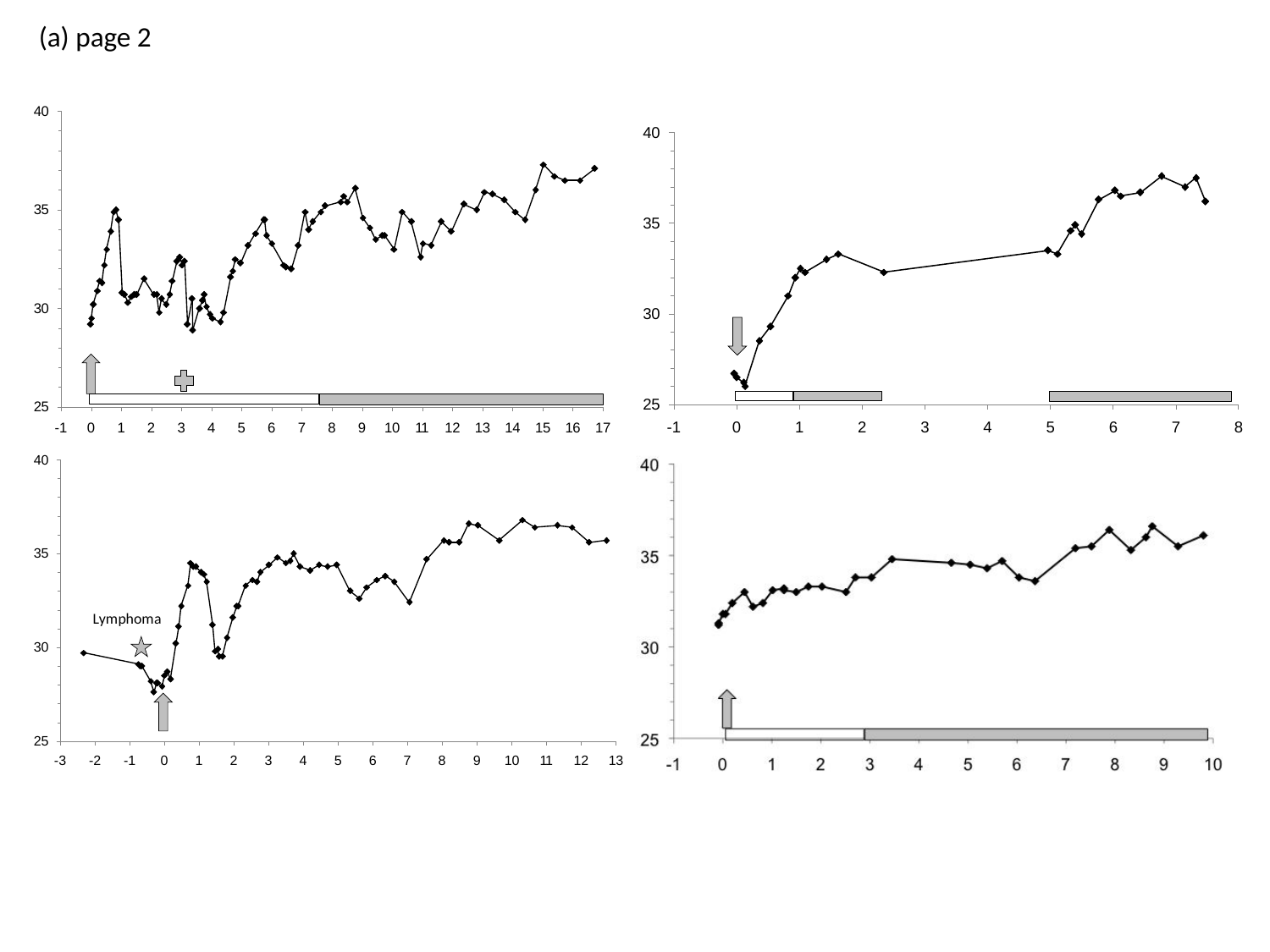

(a) page 2

## Slide 5
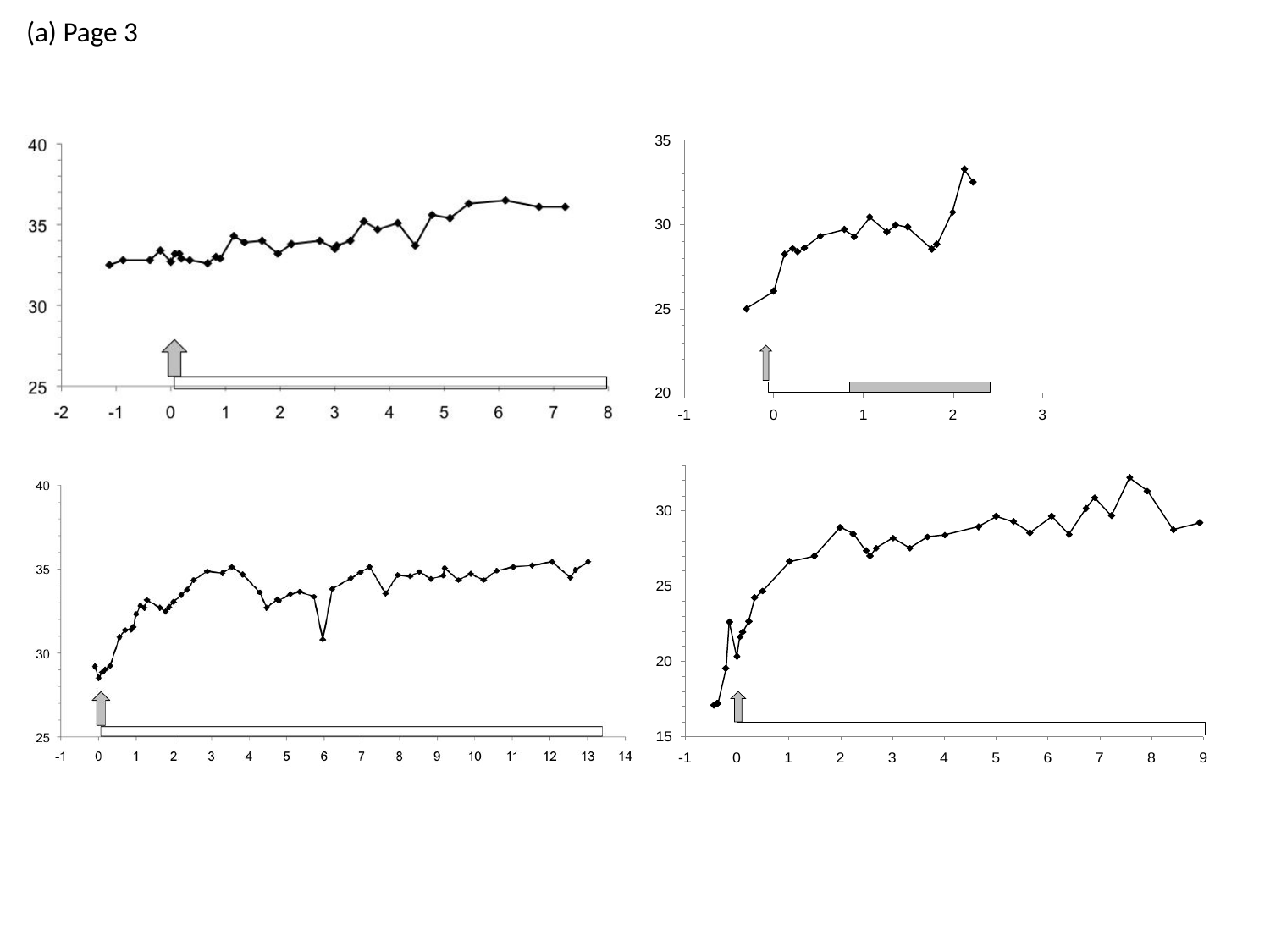

(a) Page 3

## Slide 6
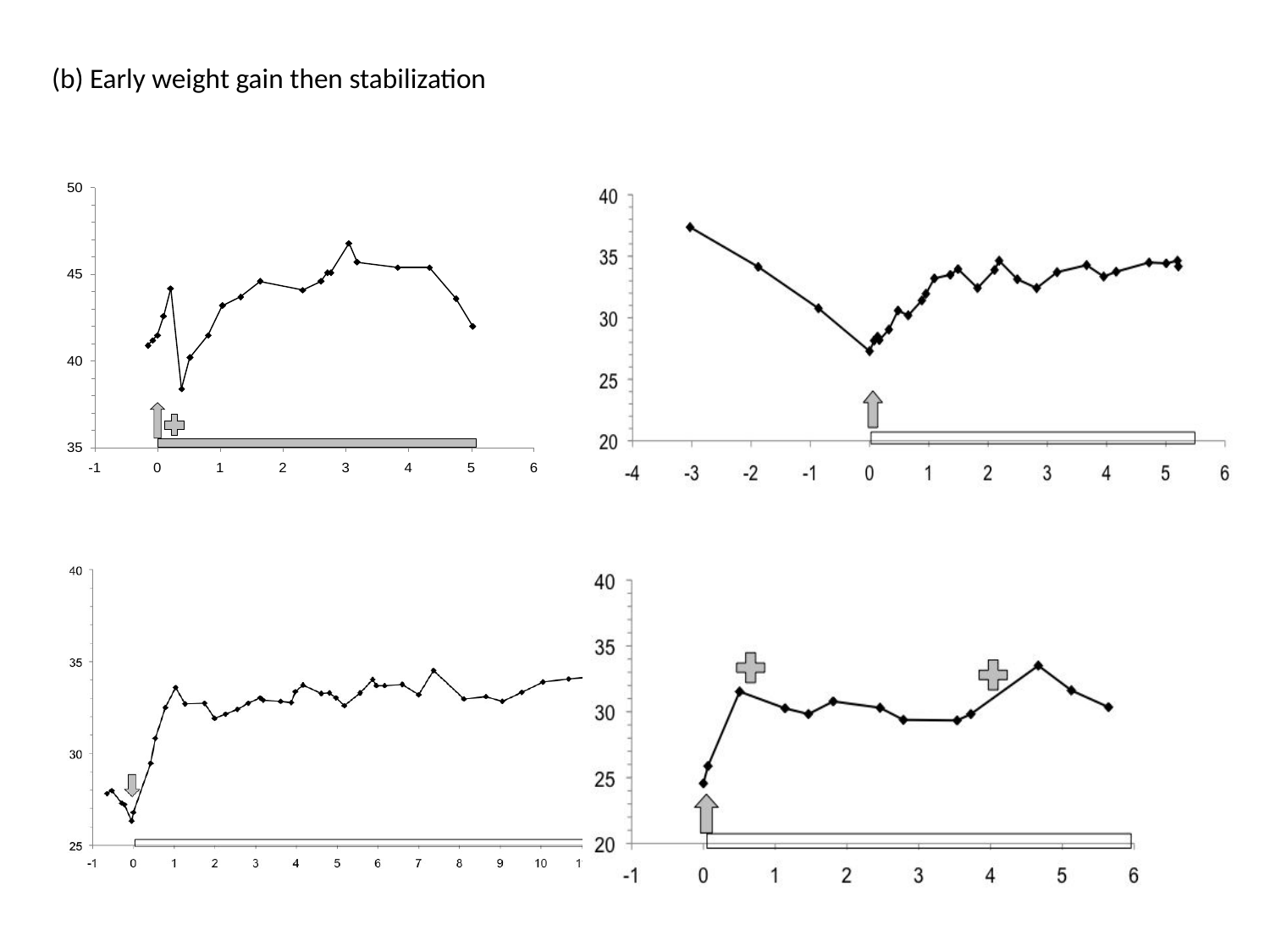

(b) Early weight gain then stabilization

## Slide 7
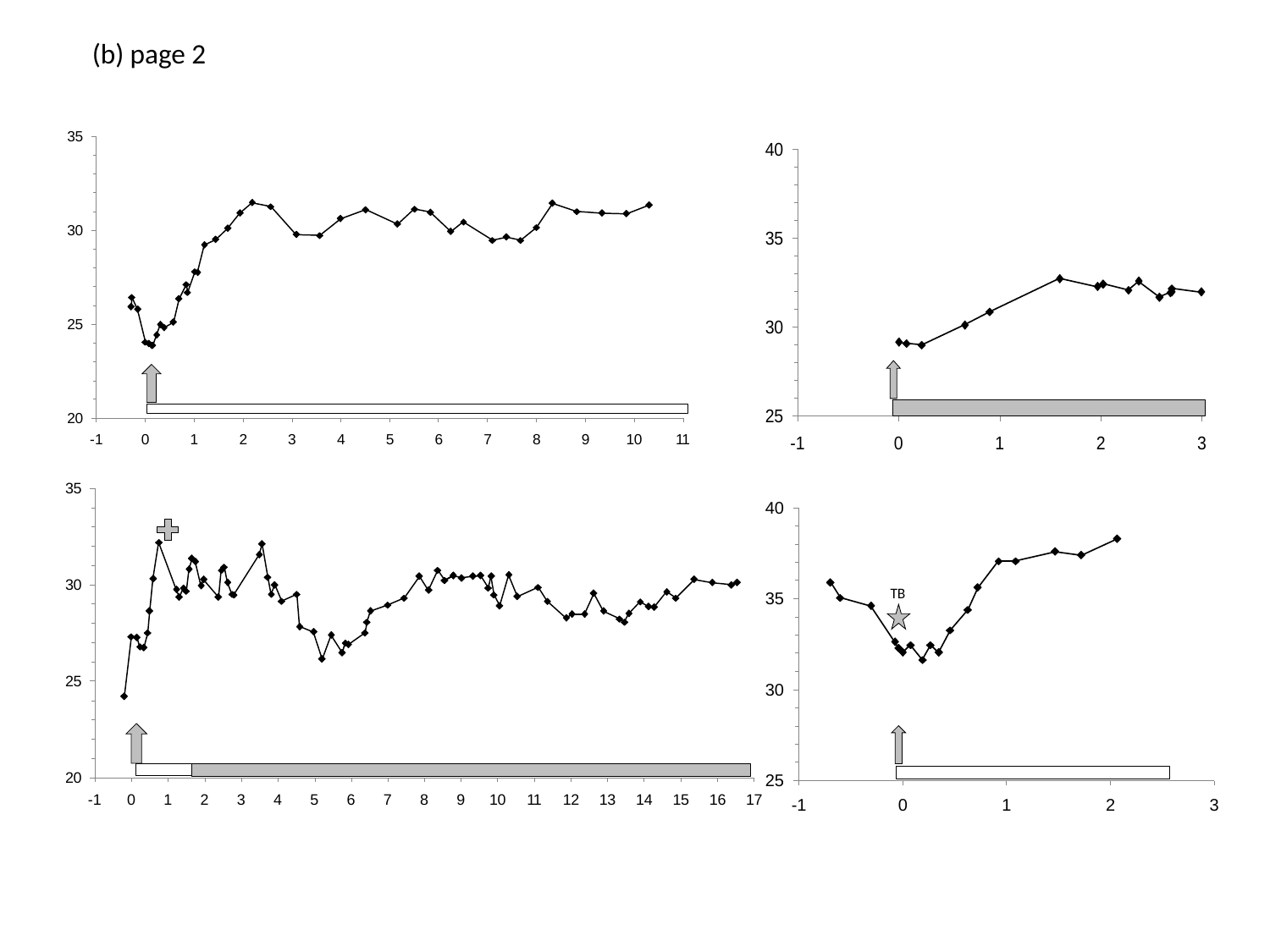

(b) page 2

## Slide 8
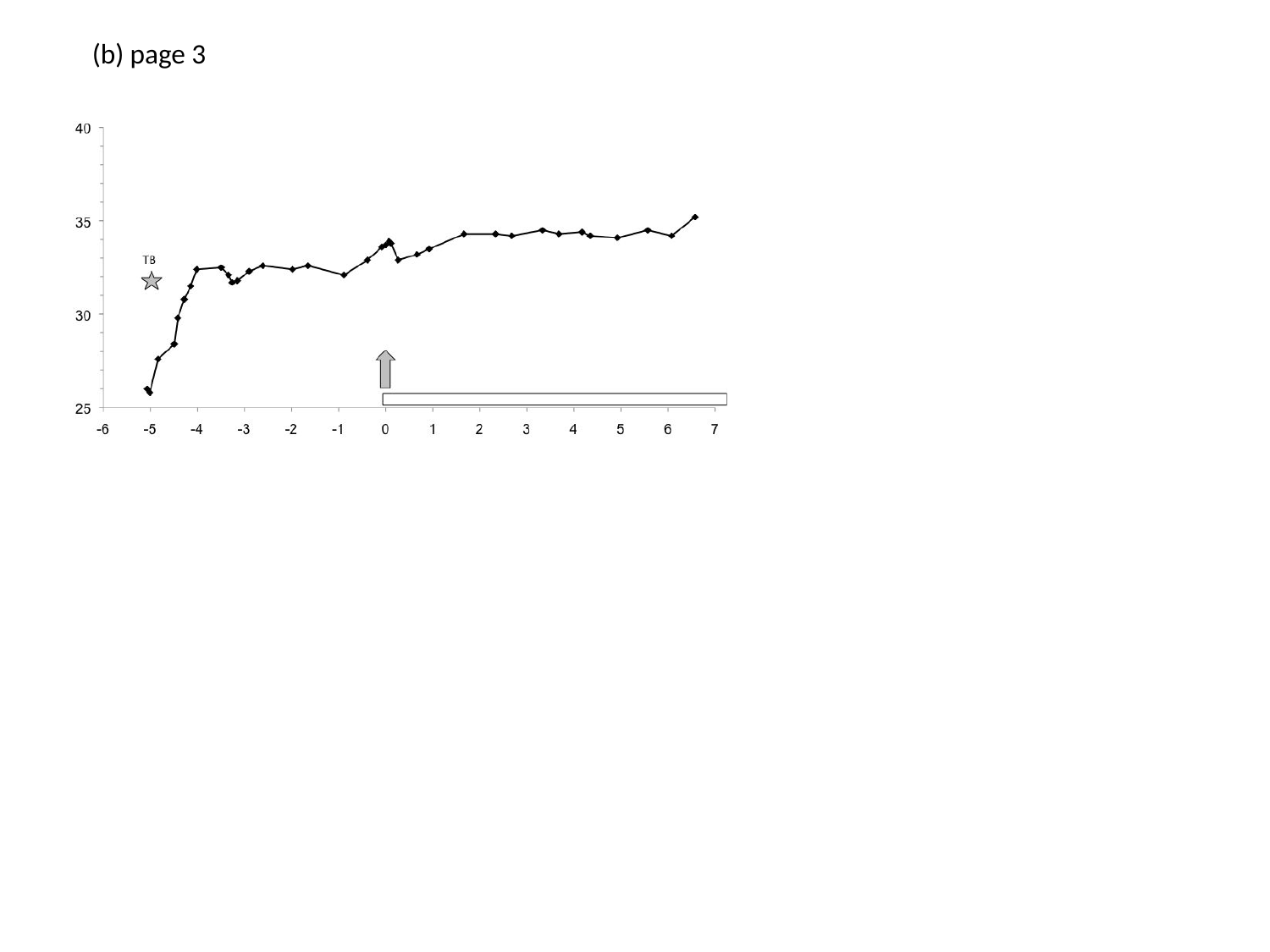

(b) page 3

## Slide 9
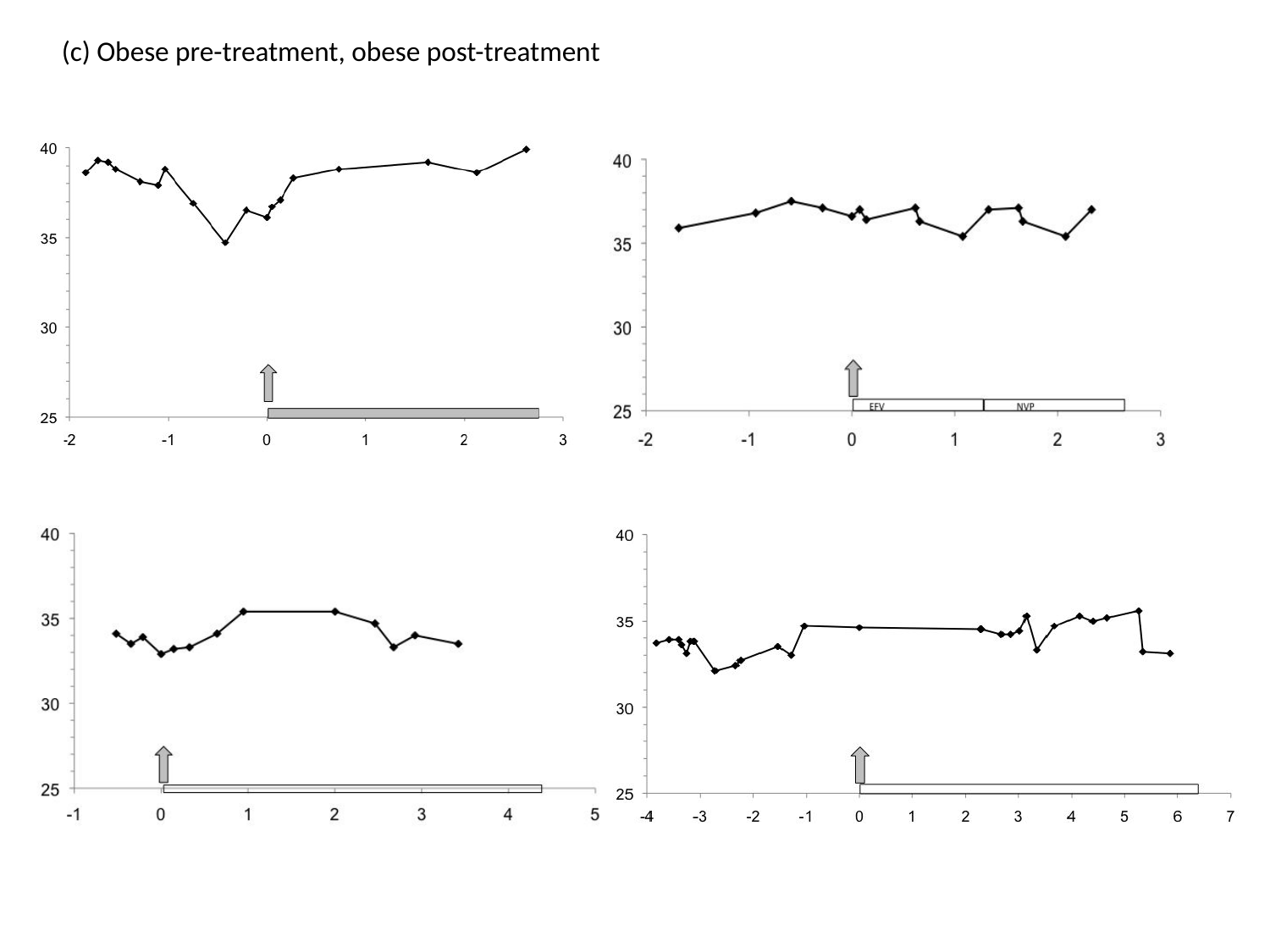

(c) Obese pre-treatment, obese post-treatment

## Slide 10
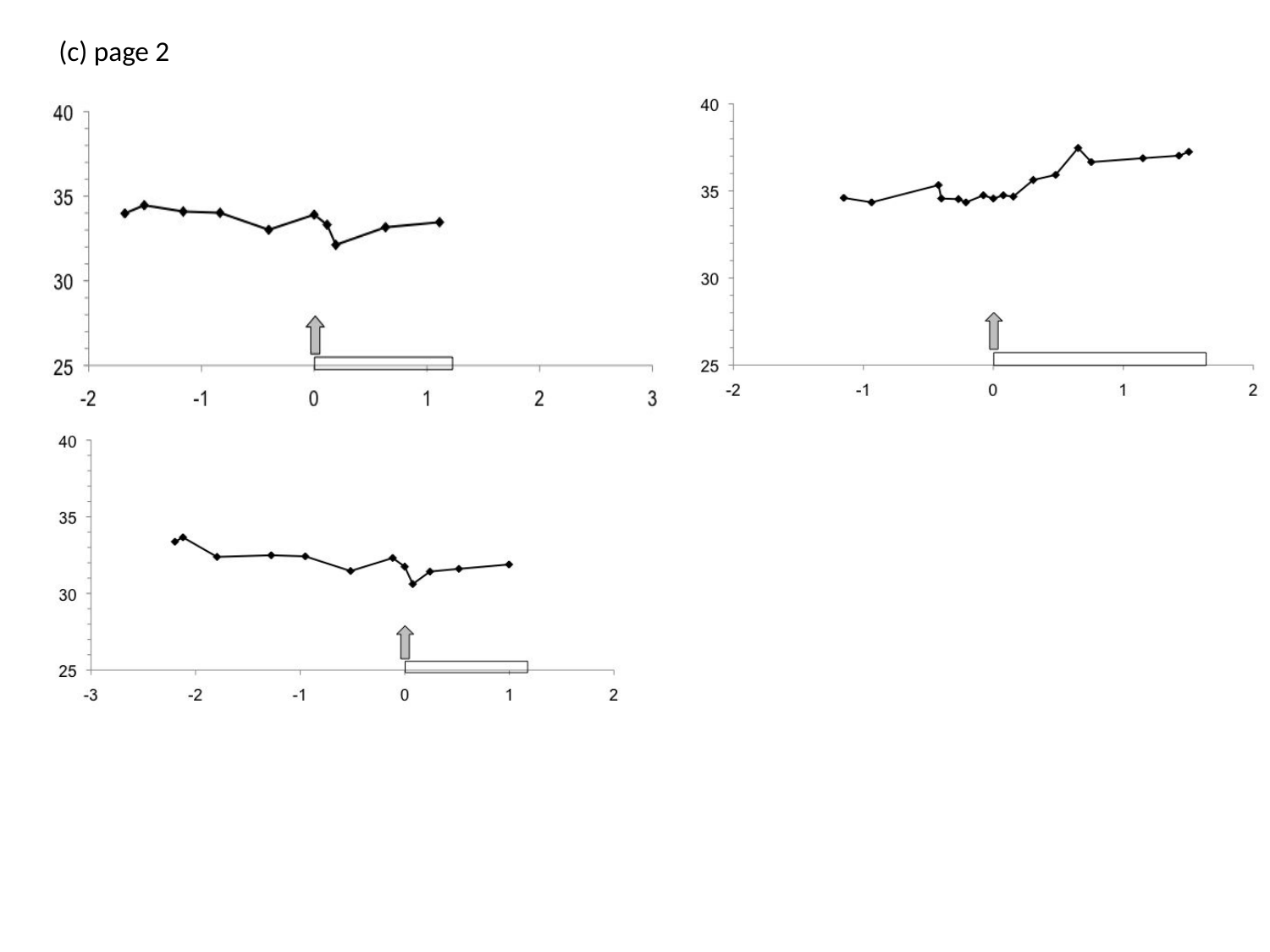

(c) page 2

## Slide 11
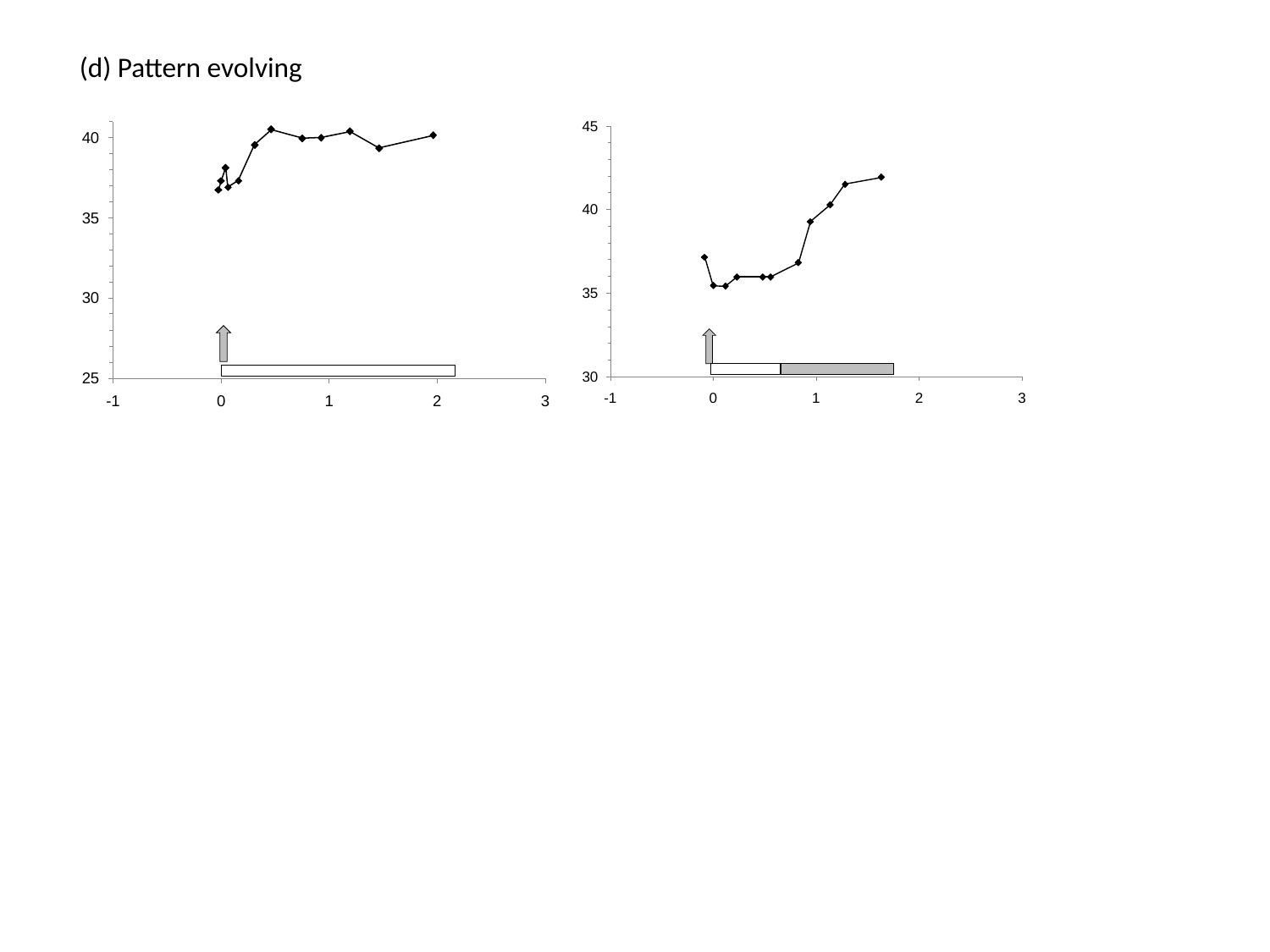

(d) Pattern evolving
